# Prognostic importance of an indicator related to systemic inflammation and insulin resistance in patients with gastrointestinal cancer: a prospective study

**DOI:** 10.3389/fonc.2024.1394892

**Published:** 2024-12-02

**Authors:** Guo-Tian Ruan, Jin-Yu Shi, Hai-Lun Xie, He-Yang Zhang, Hong Zhao, Xiao-Yue Liu, Yi-Zhong Ge, Xiao-Wei Zhang, Ming Yang, Li-Chen Zhu, Han-Ping Shi

**Affiliations:** ^1^ Department of Gastrointestinal Surgery, Beijing Shijitan Hospital, Capital Medical University, Beijing, China; ^2^ Department of Clinical Nutrition, Beijing Shijitan Hospital, Capital Medical University, Beijing, China; ^3^ National Clinical Research Center for Geriatric Diseases, Xuanwu Hospital, Capital Medical University, Beijing, China; ^4^ Key Laboratory of Cancer Foods for Special Medical Purpose (FSMP) for State Market Regulation, Beijing, China; ^5^ Laboratory for Clinical Medicine, Capital Medical University, Beijing, China

**Keywords:** systemic inflammation, insulin resistance, CTI, overall survival, gastrointestinal cancer

## Abstract

**Background:**

Systemic inflammation (SI) and insulin resistance (IR) are correlated to the progression of gastrointestinal (GI) cancer. Therefore, this study aimed to analyze the potential clinical value of the C-reactive protein-triglyceride-glucose index (CTI) in relation to SI and IR in patients with GI cancer.

**Methods:**

This prospective cohort study included patients with GI cancer. Patient data were collected from Fujian Cancer Hospital as an external validation cohort. Prognostic AUC, time-dependent ROC curve, C-index, and calibration curve analyses were used to predict the efficacy and accuracy of CTI survival prediction. Multivariate survival analysis was performed to evaluate the potential prognostic value of the CTI. Multiple logistic regression was performed to evaluate the relationship between the CTI and 90-day and 180-day mortalities.

**Results:**

We divided 1520 patients with GI cancer (mean age, 60.39 ± 11.3 years; male sex, 67%) into a training cohort and internal validation cohort; the external validation cohort included 476 patients. Prognostic AUC, time-dependent ROC curve, C-index, and calibration curve analyses of all cohorts indicated that the CTI could reliably and accurately predict the short- and long-term survival outcomes of patients with GI cancer. Multivariate survival analysis showed that for each standard deviation increase in the CTI, the risk of death increased by 32%, 21%, and 40% in the training, internal validation, and external validation cohorts, respectively. A high CTI was correlated to worse survival in patients with GI cancer (training cohort, hazard ratio [HR]=1.67, 95% confidence interval [CI]=1.35–2.08; internal validation cohort, HR=1.51, 95% CI=1.07–2.14, and external validation cohort, HR=1.59, 95% CI=1.18–2.13). In different tumor subgroups, a high CTI predicted worse survival outcomes for upper GI cancer (HR=1.54, 95% CI=1.18–2.01) and lower GI cancer (HR=1.98, 95% CI=1.36–2.86). Multivariate logistic regression analysis showed that a high CTI was positively correlated with 90-day (odds ratio [OR]=3.25, 95% CI=1.75–6.23) and 180-day mortalities (OR=2.66, 95% CI=1.72–4.15).

**Conclusions:**

The CTI is related to SI and IR and can predict the short- and long-term prognosis of patients with GI cancer. Evaluation of the CTI could provide clinicians with an effective tool for predicting the prognosis of patients with GI cancer.

**Clinical trial registration:**

https://www.chictr.org.cn/showproj.html?proj=31813, identifier ChiCTR1800020329.

## Background

Gastrointestinal (GI) cancer is a malignant tumor that occurs in the gastrointestinal tract and digestive organs (1). Several types of GI cancer share a common endoderm origin (2). The most common GI cancers include esophageal cancer (EC), gastric cancer (GC), and colorectal cancer (CRC) (3). Globally, GI cancers account for approximately 19% of all cancer cases and 22.5% of cancer-related deaths (4). Owing to the difficulty in diagnosis and high incidence of GI tumors, their treatment remains a challenge worldwide. At present, interventional measures for GI tumors include surgical treatment, preoperative neoadjuvant therapy, and postoperative adjuvant therapy, among which surgical treatment is still the most effective intervention for patients with GI tumors (5, 6); however, the therapeutic effect shows that we are still far from an effective cure. The early symptoms of most patients with GI tumors are not obvious, and most patients have local infiltration or distant metastasis at the time of their first visit; therefore, they cannot undergo radical surgery (7). These phenomena may be related to the tumor characteristics of patients with GI cancer. Hence, exploring and developing new prognostic tools will be helpful in evaluating the status of patients with GI and monitoring their short- and long-term survival outcomes.

Recently, some studies have reported a new index related to systemic inflammation (SI) and insulin resistance (IR), the C-reactive protein (CRP)-triglyceride-glucose index (CTI), which can predict the survival of patients with cancer and the poor prognosis of cancer patients with a high CTI (8). SI and IR play important roles in the occurrence and progression of cancer (9–11). SI is not only the seventh most common marker of host-tumor interactions in patients with cancer but is also one of the enabling characteristics of cancer (10–13). CRP is considered key to SI and proinflammatory cytokine (14). Elevated CRP levels indicate a systemic inflammatory response (15). Increased CRP levels in patients with GI cancer are positively relevant to larger tumor size, metastasis, and mortality (16–19). IR differs from type 2 diabetes mellitus (T2D) in patients with cancer, as it is a key constituent part of metabolic syndrome. The morbidity and mortality rates of patients with IR are increasing (20), and there is an evidence that IR is correlated to the risk of cancer, including CRC (21). Additionally, IR occurs not only in patients with cancer but also in those with cachexia (22). Patients with cancer are exposed to proinflammatory cytokines and insulin growth factor-binding proteins, leading to cancer cachexia and IR (23, 24). Thus, we can infer that SI and IR are closely related to cancer progression. In previous studies, a simple and feasible IR index, the triglyceride glycemic index (TyG), has been reported to be associated with tumorigenesis and progression (25–27). Coincidentally, the CTI includes both SI and IR. SI and IR are inseparable. A pan-cancer study found that IR is associated with SI in patients (21), and Xia et al. found that inflammation plays a key role in the progression of IR via the immune system (28). IR and CRP levels were correlated to weight loss in 10 male patients with lung cancer (29). Therefore, we have reason to construct an index related to SI and IR in patients with GI cancer (including EC, GC, and CRC), which not only reflects the state of inflammation and IR but also predicts the prognosis of patients. This study was to explore and comprehensively analyze the potential prognostic value of SI- and IR-related CTI in patients with GI cancer.

## Methods

### Study design and population

In this prospective, multicenter, and cross-sectional study, the data were from the “Investigation on Nutrition Status and its Clinical Outcome of Common Cancers” (INSCOC) study, which included patients with cancer in multiple medical centers in China from 2013 to 2021 (30–34). The cohort study included men and women aged >18 years who were pathologically diagnosed with cancer and had autonomous consciousness without communication disorders. This study was approved by various hospital ethics committees and was conducted in accordance with the Declaration of Helsinki. All participants provided written informed consent.

A total of 5221 patients with cancer were included in the cancer cohort study. Then, 1520 patients with GI cancer were randomly classified into two groups at a ratio of 7:3 to form a training cohort (1064) and an internal validation cohort (456). To further validate the reliability of the results, we collected data from 476 patients with GI cancer from Fujian Cancer Hospital in China as our external validation cohort (**Supplementary Figure S1**).

### Data collection

The data collected in this cohort included basic patient data, living habits, complications, tumor-related data, laboratory examination data, anthropometric data, questionnaire data, and nutrition-related data. Basic data included age and sex. Living habits included alcohol consumption (yes/no) and smoking status (yes/no). Complications included diabetes (yes/no), hypertension (yes/no), and coronary heart disease (CHD) (yes/no). Tumor-related data included tumor stage, tumor type, surgical treatment status (yes/no), chemotherapy treatment status (yes/no), and radiotherapy treatment status (yes/no). The laboratory data included CRP, fasting blood glucose(FBG), and triglyceride (TG) levels. Anthropometric data included body mass index (BMI) and triceps skinfold thickness (TSF). The questionnaire survey data included the Karnofsky Performance Status (KPS) and Eastern Cooperative Oncology Group Performance Status (ECOG PS). Nutrition-related variables included the Patient-Generated Subjective Global Assessment (PGSGA) and nutritional intervention (yes/no).

Trained clinicians, nurses, and clinical dietitians conducted the questionnaires. The data were then reviewed and uploaded to the system. Anthropometric data were measured by clinicians, and the BMI was calculated on the basis of the square ratio of the patient’s weight (kg) to height (m^2^). TSF was defined as the measurement of the skinfold thickness (cm) on the dorsal midpoint of the upper arm with the patient in an upright position. Blood samples were collected without treatment within 48 hours of admission. The patients fasted for at least 8 hours before blood collection, and the collected blood samples were tested in the laboratory. The TyG index was calculated as ln [TG (mg/dl)× FBG (mg/dl)]/2. The CTI is defined as the index of inflammatory insulin and calculated as follows: CTI=0.412×ln (CRP)+TyG (8). Correlations between CTI and CRP, and CTI and TyG are shown in **Supplementary Figure S2**.

### Study outcomes

The primary endpoint of this study was overall survival, which was defined as the time from admission to the last follow-up or death. Additionally, low KPS, high ECOG PS, 90-day and 180-day mortalities were the secondary endpoints, and the 90-day and 180-day mortalities were defined as the time from enrollment to death at 90 and 180 days, respectively.

### Statistical analysis

The data of continuous variables satisfying a normal distribution are reported by mean ± standard deviation (SD), and these data were compared between the groups using the Student t-test. Continuous variables that were not normally distributed re expressed as median and interquartile range, and these data were compared between the groups performing the Wilcoxon test. The data of classified variables are displayed as quantity and percentage, and the chi-square test was used to compare these data between the groups. The correlation between the CTI and different parameters was analyzed using Pearson analysis. We used maximum selection rank statistics to select the tangent point of the CTI in the training cohort. The tangent value of the CTI in GI cancer was 4.65 (**Supplementary Figure S3**). The construction of the risk score is based on the product of CTI value and risk coefficient. Multivariate survival analyses of data from the training, internal, and external validation cohorts were performed. Prognostic area under the curve (AUC), time-dependent receiver operating characteristic (ROC) curve and calibration curve analyses were conducted to compare the potential prognostic prediction ability of the CTI in patients with GI cancer. To further eliminate the interference of potentially confounding variables, multivariate survival analysis was performed using multiple adjustment models: model 1, unadjusted; model 2, adjusted for age, sex, BMI, and tumor stage; model 3, adjusted for model 2+tumor type, surgery, radiotherapy, chemotherapy, smoking, drinking, diabetes, hypertension, CHD, KPS, ECOG PS, PGSGA, and nutritional intervention; and model 4, adjusted for model 3+TSF. Hazard ratios (HRs) and 95% confidence intervals (CIs) were performed to evaluate survival. Mediating effect analysis was performed to investigate the contribution of potential variables to the prognosis based on the CTI. We examined the potential mediating role of ECOG PS and KPS in the relationship between CTI and OS. The value of the mediation proportion represents the mediation strength of the variable. We performed multiple logistic regression analyses to investigate the relationship between potential factors and low KPS, high ECOG PS, 90-day, and 180-day mortalities, adjusted using model 4. Odds ratios (ORs) and 95% CIs were used in the logistic regression analysis. To rule out causal inversion, we conducted a sensitivity analysis, excluding patients who died within 6 months from multivariate survival analysis, and corrected model selection to model 4.

All double-tailed *P*-values <0.05 indicated the result had a statistical significance, and all statistical analyses were carried out using R software (version 4.1.1; R Core Team).

## Results

### Baseline characteristics

The average age of the 1520 patients was 60.39 ± 11.33 years, including 1019 men (67.0%). In the training cohort, the mean age of the 1064 patients with GI cancer was 60.61 ± 11.31 years, including 724 men (68.0%). In the internal validation cohort, the mean age of the 456 patients with GI cancer was 59.88 ± 11.38 years, including 295 men (64.7%). In the external validation cohort, the mean age of the 476 patients with GI cancer was 58.19 ± 11.81 years, including 335 men (70.4%). In these cohorts, there were more patients with advanced tumor stages, more patients undergoing surgery, and more patients with malnutrition than their counterparts. The baseline characteristics of the three cohorts are listed in **Table 1**.

**Table 1:** Baseline characteristics.

Variables	Training cohort (n=1064)	Internal validation cohort (n=456)	External validation cohort (n=476)
Sex (%)			
male	724 (68.0)	295 (64.7)	335 (70.4)
female	340 (32.0)	161 (35.3)	141 (29.6)
Age, years (mean (SD))	60.61 (11.31)	59.88 (11.38)	58.19 (11.81)
BMI, kg/m^2 (mean (SD))	21.85 (3.52)	21.80 (3.17)	21.80 (3.04)
BMI, kg/m^2 (%)			
<24	774 (72.7)	345 (75.7)	361 (75.8)
≥24	290 (27.3)	111 (24.3)	115 (24.2)
Smoking, yes (%)	480 (45.1)	210 (46.1)	217 (45.6)
Drinking, yes (%)	290 (27.3)	126 (27.6)	108 (22.7)
Diabetes, yes (%)	105 (9.9)	34 (7.5)	42 (8.8)
Hypertension, yes (%)	218 (20.5)	84 (18.4)	84 (17.6)
CHD, yes (%)	44 (4.1)	17 (3.7)	9 (1.9)
Tumor stage (%)			
I-II	286 (26.9)	112 (24.6)	73 (15.3)
III-IV	778 (73.1)	344 (75.4)	403 (84.7)
Surgery, yes (%)	676 (63.5)	316 (69.3)	312 (65.5)
Radiotherapy, yes (%)	86 (8.1)	37 (8.1)	27 (5.7)
Chemotherapy, yes (%)	679 (63.8)	293 (64.3)	185 (38.9)
Tumor type (%)			
Upper digestive tract tumors			
EC (%)	182 (17.1)	77 (16.9)	54 (11.3)
GC (%)	402 (37.8)	153 (33.6)	211 (44.3)
Lower digestive tract tumors			
CRC (%)	480 (45.1)	226 (49.6)	211 (44.3)
Glucose, mmol/L (mean (SD))	5.76 (1.72)	5.58 (1.55)	5.46 (1.71)
TG, mmol/L (mean (SD))	1.41 (1.04)	1.41 (0.75)	1.34 (0.81)
CRP, mg/L (median (IQR))	3.29 (10.66)	3.13 (9.36)	3.55 (111.80)
TyG (mean (SD))	3.85 (0.29)	3.85 (0.28)	3.81 (0.27)
CTI (mean (SD))	4.43 (0.81)	4.39 (0.75)	4.67 (0.54)
ECOG (%)			
<2	939 (88.3)	413 (90.6)	152 (31.9)
≥2	125 (11.7)	43 (9.4)	324 (68.1)
KPS (mean (SD))	84.56 (13.76)	84.65 (13.44)	83.53 (5.67)
PGSGA (mean (SD))	7.52 (5.12)	7.16 (4.75)	6.12 (3.85)
Nutrition intervention (%)	356 (33.5)	130 (28.5)	55 (11.6)
TSF, cm (mean (SD))	14.77 (9.47)	15.85 (9.98)	13.22 (6.59)
LOS, days (mean (SD))	12.03 (10.84)	12.95 (12.08)	11.77 (7.32)
Hospital costs, yuan (mean (SD))	32356.40 (61993.26)	31559.94 (41039.72)	28606.86 (25377.47)

BMI, body mass index; CHD, coronary heart disease; EC, esophagus cancer; GC, gastric cancer; CRC, colorectal cancer; TG, triglyceride; CTI, CRP-TyG index; CRP, C-reactive protein; TyG, triglyceride-glucose index; KPS, karnofsky performance status; ECOG PS, eastern cooperative oncology group performance status; PGSGA, Patient Generated Subjective Global Assessment; TSF, triceps skinfold thickness; LOS, length of stay.

### Construction and verification of the potential survival predictive value of the CTI in patients with GI tumors in all cohorts

#### Calibration curve

We performed calibration curves to explore the survival predictive consistency of CTI in patients with GI cancer. The CTI had good predictive consistency in all cohorts (**Supplementary Figure S4**).

#### Survival prediction ability

**Figure 1:**
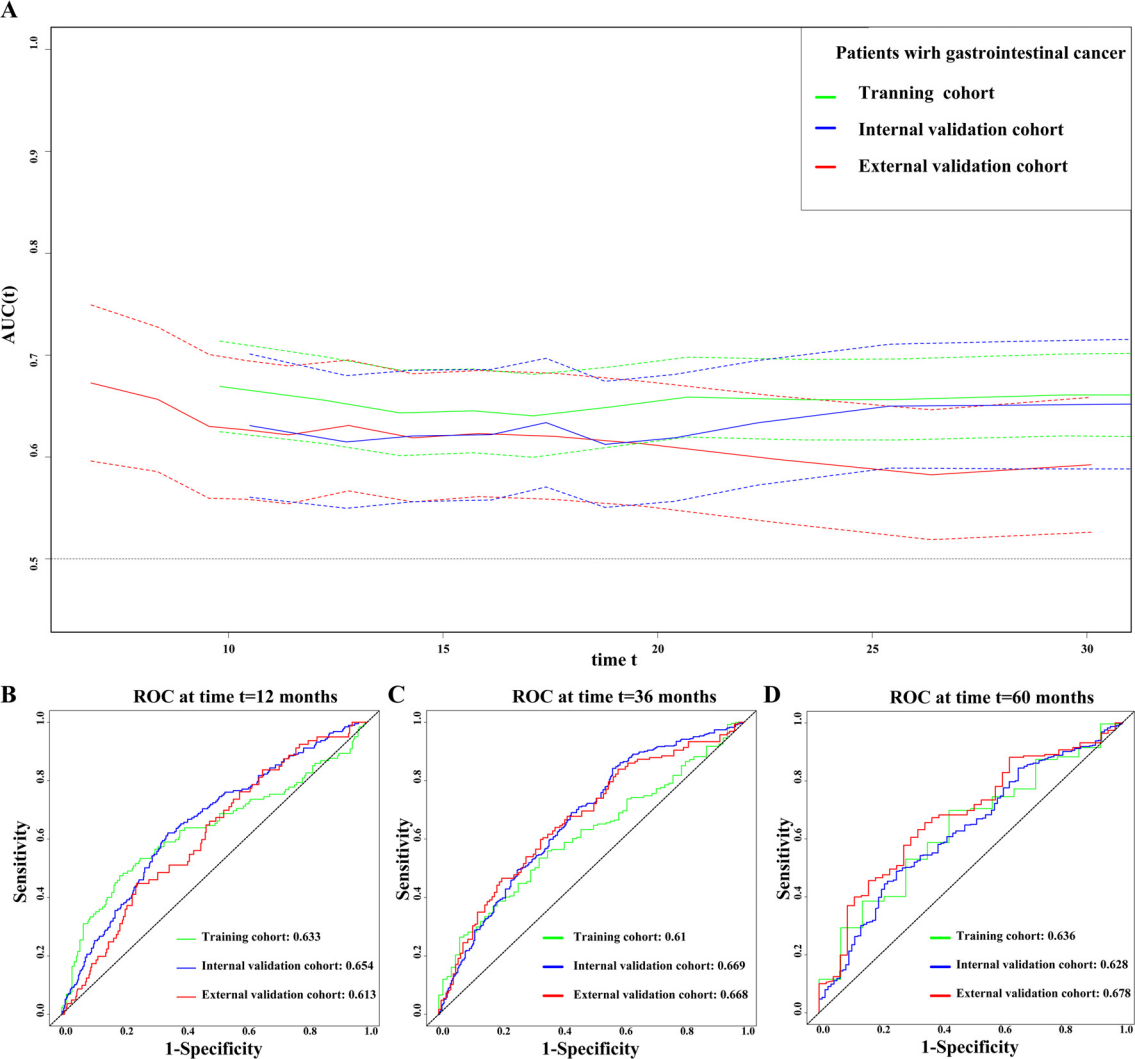
Evaluate the ability of CTI to predict the prognosis of patients with GI cancer in all cohorts. **(A)** Prognostic AUC curve; **(B)** 1-year time-dependent ROC curve; **(C)** 3-year time-dependent ROC curve; **(D)** 5-year time-dependent ROC curve. The green line represents the training cohort, the blue line represents the internal validation cohort, and the red line represents the external validation cohort. GI, gastrointestinal; CTI, C-reactive protein-triglyceride glucose index; AUC, area under the curve; ROC, receiver operating characteristic.

We used the prognostic AUC and ROC curve to explore the prognostic value of the CTI in patients with GI cancer. First, we found that the CTI had a high AUC in the training, internal validation, and external validation cohorts, both in short-term and long-term viability predictions (**Figure 1A**). Second, we performed survival AUC analysis and found that the CTI had a high and good survival prediction ability in the 1-year (training cohort: AUC=0.633; internal validation cohort: AUC=0.654, and external validation cohort: AUC=0.613), 3-year (training cohort: AUC=0.610; internal validation cohort: AUC=0.669, and external validation cohort: AUC=0.668), and 5-year ROC curves (training cohort: AUC=0.636; internal validation cohort: AUC=0.628, and external validation cohort: AUC=0.678) (**Figures 1B–D**). The C-index indicated that the CTI had a good and consistent survival prediction ability in the training (0.620, 95% CI=0.59–0.65), internal validation (0.600, 95% CI=0.56–0.65), and external validation cohorts (0.608, 95% CI=0.56–0.65).

#### Survival analysis

**Figure 2:**
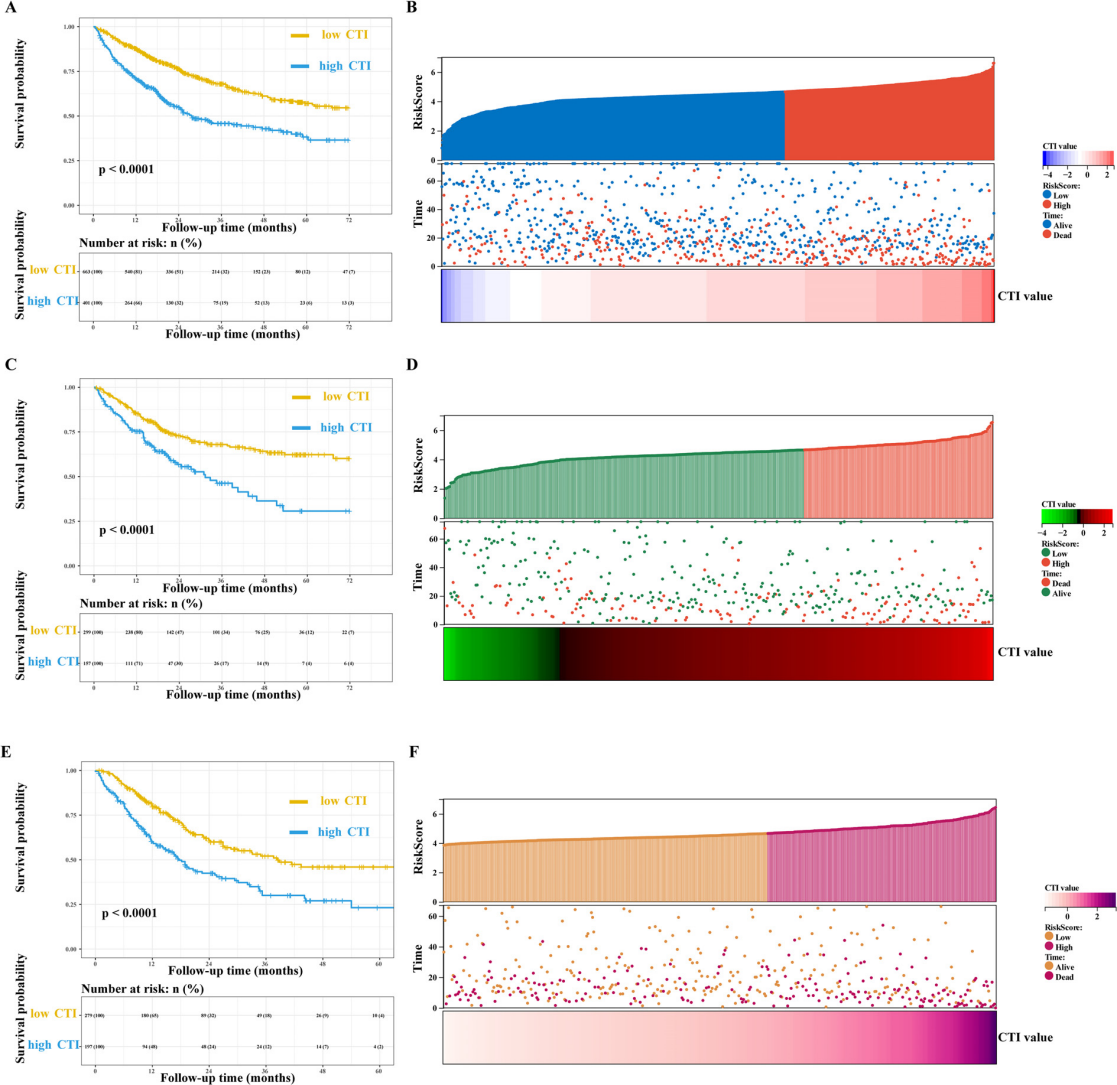
Survival curve and prognostic risk score map of CTI in patients with GI cancer. **(A, B)** Training cohort, **(A)**, survival curve, **(B)**, prognostic risk score map; In the survival curve, the yellow line represents low CTI and the blue line represents high CTI; In the prognostic risk score map, red represents the high-risk score, blue represents the low-risk score, and red represents death and blue represents the living in the survival status map. **(C, D)** Internal validation cohort, **(C)**, survival curve, **(D)**, prognostic risk score map; In the prognostic risk score map, red represents the high-risk score, green represents the low-risk score, and red represents death and green represents the living in the survival status map. **(E, F)** External validation cohort, **(E)**, survival curve, **(F)**, prognostic risk score map; In the prognostic risk score map, purple represents the high-risk score, orange represents the low-risk score, and purple represents death and orange represents the living in the survival status map. GI, gastrointestinal; CTI, C-reactive protein-triglyceride glucose index.

Multivariate survival analysis showed that the CTI had good and consistent short- and long-term predictive abilities in patients with GI cancer. The death risks of each additional SD of CTI value in patients with GI cancer increased by 32% (training cohort, adjusted model 4: HR=1.32, 95% CI=1.17–1.48), 21% (internal validation cohort, adjusted model 4: HR=1.21, 95% CI=1.03–1.43), and 40% (external validation cohort, adjusted model 4: HR=1.40, 95% CI=1.21–1.61). Additionally, the death risk increased in patients with a high CTI (training cohort, adjusted model 4: HR=1.67, 95% CI=1.35–2.08; internal validation cohort, adjusted model 4: HR=1.51, 95% CI=1.07–2.14; and external validation cohort, adjusted model 4: HR=1.59, 95% CI=1.18–2.13) (**Figure 2**, **Table 2**, **Supplementary Figure S5**).

**Table 2:** Survival analyses.

Variables	OS (model 1) ^a^		OS (model 2) ^b^		OS (model 3) ^c^		OS (model 4) ^d^	
	Crude HR(95%CI)	Crude P	Adjusted HR (95%CI)	Adjusted P	Adjusted HR(95%CI)	Adjusted P	Adjusted HR(95%CI)	Adjusted P
Training cohort								
as continues (per SD)	1.44 (1.29-1.61)	<0.001	1.46 (1.31-1.63)	<0.001	1.31 (1.16-1.47)	<0.001	1.32 (1.17-1.48)	<0.001
By cut-off								
CTI<4.65	ref.		ref.		ref.		ref.	
CTI≥4.65	2.02 (1.65-2.46)	<0.001	2.04 (1.67-2.49)	<0.001	1.66 (1.34-2.06)	<0.001	1.67 (1.35-2.08)	<0.001
By Interquartile								
Q1(<4.11)	ref.		ref.		ref.		ref.	
Q2(4.11-4.46)	1.60 (1.17-2.18)	0.003	1.65 (1.21-2.25)	0.002	1.37 (1.00-1.88)	0.049	1.39 (1.01-1.90)	0.042
Q3(4.46-4.91)	1.87 (1.38-2.54)	<0.001	1.98 (1.45-2.69)	<0.001	1.76 (1.28-2.41)	<0.001	1.73 (1.26-2.38)	0.001
Q4(>4.91)	2.73 (2.03-3.65)	<0.001	2.81 (2.10-3.78)	<0.001	2.05 (1.50-2.81)	<0.001	2.09 (1.53-2.86)	<0.001
p for trend		<0.001		<0.001		<0.001		<0.001
Internal validation cohort								
as continues (per SD)	1.36 (1.17-1.59)	<0.001	1.38 (1.18-1.62)	<0.001	1.21 (1.03-1.43)	0.02	1.21 (1.03-1.43)	0.02
By cut-off								
CTI<4.65	ref.		ref.		ref.		ref.	
CTI≥4.65	1.99 (1.46-2.72)	<0.001	2.06 (1.50-2.82)	<0.001	1.52 (1.07-2.14)	0.018	1.51 (1.07-2.14)	0.018
By Interquartile								
Q1(<4.11)	ref.		ref.		ref.		ref.	
Q2(4.11-4.46)	1.12 (0.69-1.80)	0.648	1.12 (0.69-1.81)	0.647	1.28 (0.79-2.10)	0.318	1.28 (0.79-2.10)	0.318
Q3(4.46-4.91)	1.23 (0.78-1.93)	0.37	1.25 (0.79-1.96)	0.337	1.16 (0.73-1.83)	0.533	1.16 (0.73-1.83)	0.532
Q4(>4.91)	2.64 (1.76-3.96)	<0.001	2.72 (1.81-4.10)	<0.001	1.88 (1.20-2.94)	0.006	1.88 (1.20-2.94)	0.006
p for trend		<0.001		<0.001		0.014		0.014
External validation cohort								
as continues (per SD)	1.48 (1.30-1.69)	<0.001	1.48 (1.30-1.68)	<0.001	1.36 (1.18-1.57)	<0.001	1.40 (1.21-1.61)	<0.001
By cut-off								
CTI<4.65	ref.		ref.		ref.		ref.	
CTI≥4.65	1.93 (1.47-2.53)	<0.001	1.89 (1.44-2.47)	<0.001	1.55 (1.16-2.08)	0.003	1.59 (1.18-2.13)	0.002
By Interquartile								
Q1(<4.11)	ref.		ref.		ref.		ref.	
Q2(4.11-4.46)	0.84 (0.52-1.34)	0.454	0.87 (0.54-1.39)	0.555	0.88 (0.55-1.43)	0.611	0.90 (0.56-1.45)	0.658
Q3(4.46-4.91)	0.77 (0.47-1.26)	0.302	0.79 (0.48-1.30)	0.353	0.83 (0.50-1.38)	0.479	0.84 (0.51-1.39)	0.493
Q4(>4.91)	2.02 (1.29-3.15)	0.002	2.02 (1.29-3.16)	0.002	1.64 (1.02-2.65)	0.041	1.72 (1.06-2.77)	0.027
p for trend		<0.001		<0.001		0.005		0.003

OS, overall survival; HR, hazards ratio; CI, confidence interval; CTI, CRP-TyG index; CRP, C-reactive protein; TyG, triglyceride-glucose index; BMI, body mass index; KPS, karnofsky performance status; ECOG PS, eastern cooperative oncology group performance status; PGSGA, Patient Generated Subjective Global Assessment; TSF, triceps skinfold thickness.

a Model 1: Unadjusted.

b Model 2: Adjusted for age, sex, and BMI.

c Model 3: Adjusted for age, sex, BMI, tumor stage, tumor types, surgery, chemotherapy, radiotherapy, smoking status, alcohol consumption, KPS, ECOG PS, PGSGA, nutrition intervention, diabetes, hypertension, and coronary heart disease.

d Model 4: Adjusted for age, sex, BMI, tumor stage, tumor types, surgery, chemotherapy, radiotherapy, smoking status, alcohol consumption, KPS, ECOG PS, PGSGA, nutrition intervention, diabetes, hypertension, coronary heart disease, and TSF.

In summary, we found that the CTI can be used as a good and useful predictor of short- and long-term survival in patients with GI cancer. Therefore, we will conduct a comprehensive analysis of CTI in patients with GI cancer.

### Comprehensive analysis of the value of the CTI in patients with GI cancer based on the training cohort

#### Baseline characteristics stratified by the CTI

In the training cohort, patients with GI cancer with a high CTI were older, had more advanced tumor stages, tolerated surgery less, had a poorer quality of life, higher levels of SI, and a higher risk of malnutrition than those with a low CTI (**Supplementary Table S1**).

#### Distribution of the CTI by different subgroups

**Figure 3:**
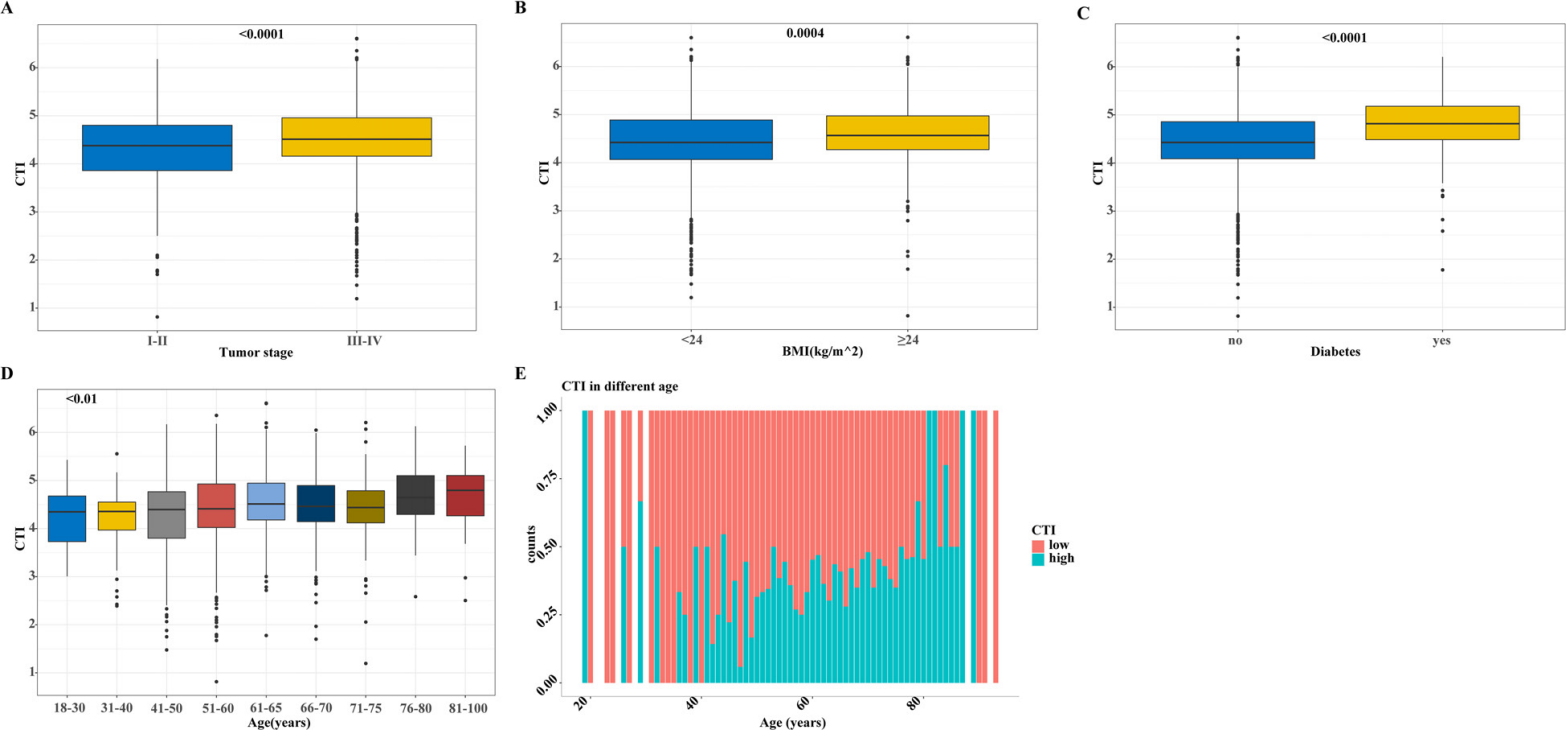
The distribution of CTI in different groups based on the training cohort. **(A-D)** The distribution of CTI as a continuous variable in different subgroups of variables, **(A)** CTI in tumor stage groups; **(B)** CTI in BMI groups; **(C)** CTI in diabetes and non-diabetes groups; **(D)** CTI in different age groups; **(E)** The distribution of CTI as a classification variable in different ages. CTI, C-reactive protein-triglyceride glucose index; BMI, body mass index.

Differences in the distribution of the CTI among the different subgroups of patients with GI cancer showed a higher BMI, advanced tumor stage, older age, and higher CTI values in patients with diabetes (**Figure 3**). We subdivided the patients according to age and the results showed that the CTI value increased with age. Interestingly, we also found that the proportion of patients with a high CTI increased with age (**Figure 3**).

#### Survival analysis of the CTI in patients with upper and lower digestive tract tumors

We analyzed and investigated the potential survival prediction value of the CTI in different tumor types. In patients with upper GI cancer (EC and GC), we found that each additional SD in the CTI increased the risk of death by 17% (adjusted model 4: HR=1.17, 95% CI=1.03–1.34). GI patients with a high CTI increased the death risk (adjusted model 4: HR=1.54, 95% CI=1.18–2.01). Additionally, our survival analysis of lower GI cancer (CRC) indicated that each additional SD in the CTI increased the death risk in patients with lower GI cancer by 49% (adjusted model 4: HR=1.49, 95% CI=1.25–1.79). GI cancer patients with a high CTI had an increased risk of death (adjusted model 4: HR=1.98, 95% CI=1.36–2.86) (**Table 3**).

**Table 3:** Survival analysis in different tumor types.

Variables	OS (model 1) ^a^		OS (model 2) ^b^		OS (model 3) ^c^		OS (model 4) ^d^	
	Crude HR(95%CI)	Crude P	Adjusted HR(95%CI)	Adjusted P	Adjusted HR(95%CI)	Adjusted P	Adjusted HR(95%CI)	Adjusted P
Upper GI cancer								
as continues (per SD)	1.30 (1.15-1.47)	<0.001	1.32 (1.16-1.49)	<0.001	1.16 (1.02-1.33)	0.027	1.17 (1.03-1.34)	0.019
By cut-off								
CTI<4.71								
CTI≥4.71	1.81 (1.42-2.32)	<0.001	1.85 (1.44-2.38)	<0.001	1.53 (1.17-2.00)	0.002	1.54 (1.18-2.01)	0.002
By Interquartile								
Q1(<4.11)								
Q2(4.11-4.46)	1.59 (1.10-2.29)	0.013	1.65 (1.14-2.39)	0.007	1.29 (0.88-1.87)	0.187	1.30 (0.89-1.89)	0.173
Q3(4.46-4.91)	1.93 (1.34-2.77)	<0.001	1.97 (1.37-2.83)	<0.001	1.69 (1.16-2.45)	0.006	1.64 (1.12-2.38)	0.01
Q4(>4.91)	2.26 (1.58-3.22)	<0.001	2.34 (1.63-3.34)	<0.001	1.61 (1.09-2.36)	0.016	1.64 (1.11-2.41)	0.013
p for trend	1.29 (1.16-1.44)	<0.001	1.30 (1.17-1.45)	<0.001	1.18 (1.05-1.33)	0.007	1.18 (1.05-1.33)	0.006
Lower GI cancer								
as continues (per SD)	1.59 (1.34-1.89)	<0.001	1.61 (1.36-1.92)	<0.001	1.49 (1.24-1.79)	<0.001	1.49 (1.25-1.79)	<0.001
By cut-off								
CTI<4.71								
CTI≥4.71	2.51 (1.79-3.51)	<0.001	2.44 (1.73-3.43)	<0.001	1.97 (1.36-2.86)	<0.001	1.98 (1.36-2.86)	<0.001
By Interquartile								
Q1(<4.11)								
Q2(4.11-4.46)	1.90 (1.05-3.43)	0.034	1.85 (1.02-3.35)	0.042	1.63 (0.89-2.99)	0.114	1.66 (0.90-3.05)	0.102
Q3(4.46-4.91)	2.17 (1.21-3.89)	0.009	2.24 (1.24-4.06)	0.007	2.22 (1.20-4.11)	0.011	2.24 (1.21-4.14)	0.010
Q4(>4.91)	4.34 (2.51-7.49)	<0.001	4.20 (2.42-7.29)	<0.001	3.37 (1.88-6.05)	<0.001	3.43 (1.91-6.18)	<0.001
p for trend	1.58 (1.35-1.86)	<0.001	1.57 (1.34-1.85)	<0.001	1.47 (1.24-1.75)	<0.001	1.48 (1.25-1.76)	<0.001

GI, gastrointestinal; OS, overall survival; HR, hazards ratio; CI, confidence interval; CTI, CRP-TyG index; CRP, C-reactive protein; TyG, triglyceride-glucose index; BMI, body mass index; KPS, karnofsky performance status; ECOG PS, eastern cooperative oncology group performance status; PGSGA, Patient Generated Subjective Global Assessment; TSF, triceps skinfold thickness.

a Model 1: Unadjusted.

b Model 2: Adjusted for age, sex, and BMI.

c Model 3: Adjusted for age, sex, BMI, tumor stage, surgery, chemotherapy, radiotherapy, smoking status, alcohol consumption, KPS, ECOG PS, PGSGA, nutrition intervention, diabetes, hypertension, and coronary heart disease.

d Model 4: Adjusted for age, sex, BMI, tumor stage, surgery, chemotherapy, radiotherapy, smoking status, alcohol consumption, KPS, ECOG PS, PGSGA, nutrition intervention, diabetes, hypertension, coronary heart disease, and TSF.

#### Sensitivity analysis

The results indicated that each additional SD in the CTI increased the death risk by 18% (adjusted model 4: HR=1.18, 95% CI=1.04–1.34). Patients with a high CTI had an increased death risk (adjusted model 4: HR=1.43, 95% CI=1.11–1.85) (**Supplementary Table S2**). The trend in the sensitivity analysis results was consistent with that of the previous analysis.

#### Subgroup analysis

**Figure 4:**
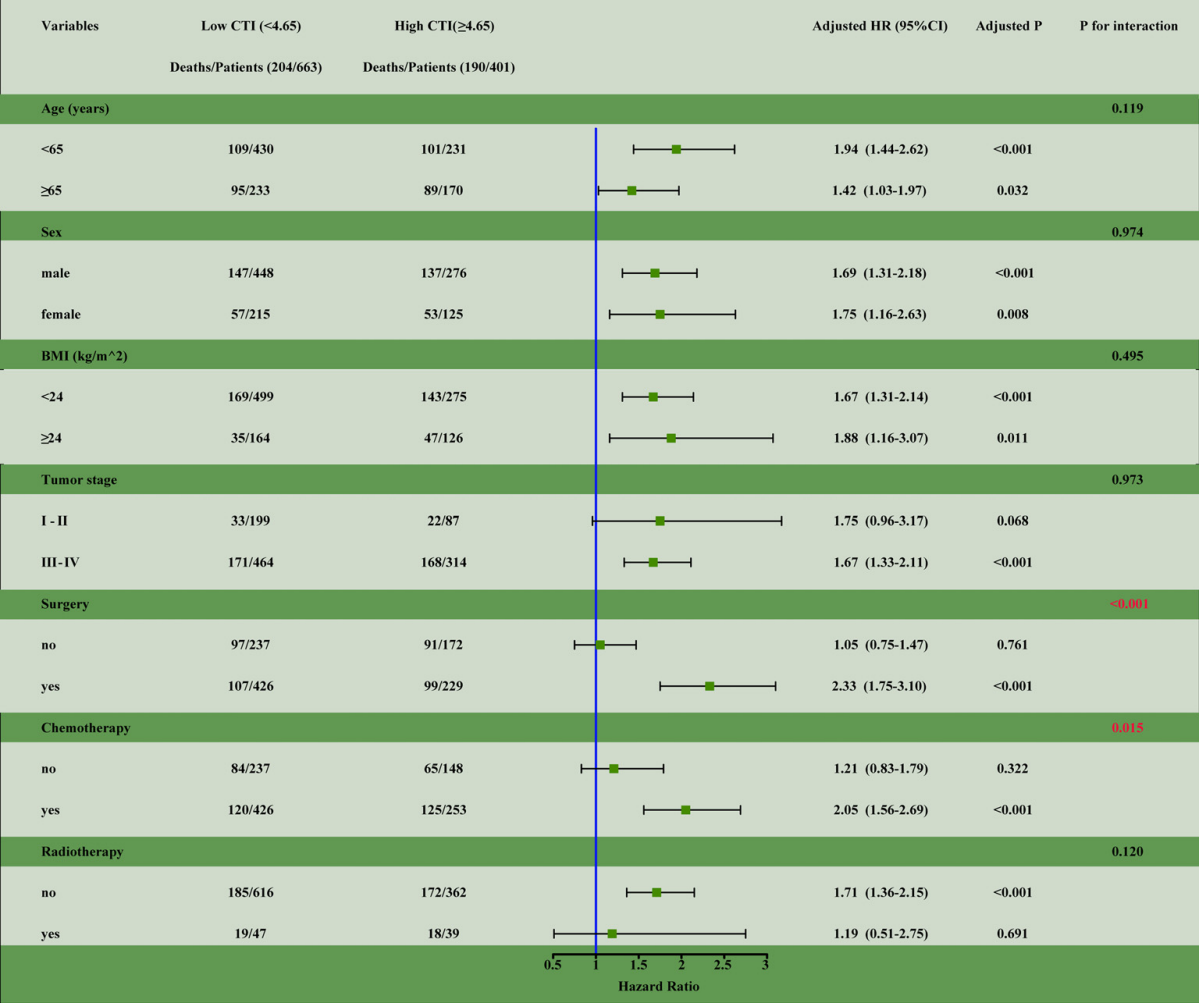
The subgroup analysis of the CTI in the training cohort of patients with GI cancer. Adjusted for age, sex, BMI, tumor stage, tumor types, surgery, chemotherapy, radiotherapy, smoking status, alcohol consumption, KPS, ECOG PS, nutrition intervention, diabetes, hypertension, coronary heart disease, and TSF. HR, hazards ratio; CI, confidence interval; CTI, CRP-TyG index; CRP, C-reactive protein; TyG, triglyceride-glucose index; BMI, body mass index; KPS, karnofsky performance status; ECOG PS, eastern cooperative oncology group performance status; TSF, triceps skinfold thickness.

We performed survival analysis for different subgroup variables. The results showed a significant interaction between a high CTI and patients undergoing surgery (interaction, *P*<0.001) and chemotherapy (interaction, *P*<0.015) (**Figure 4**).

#### Mediation analyses

**Figure 5:**
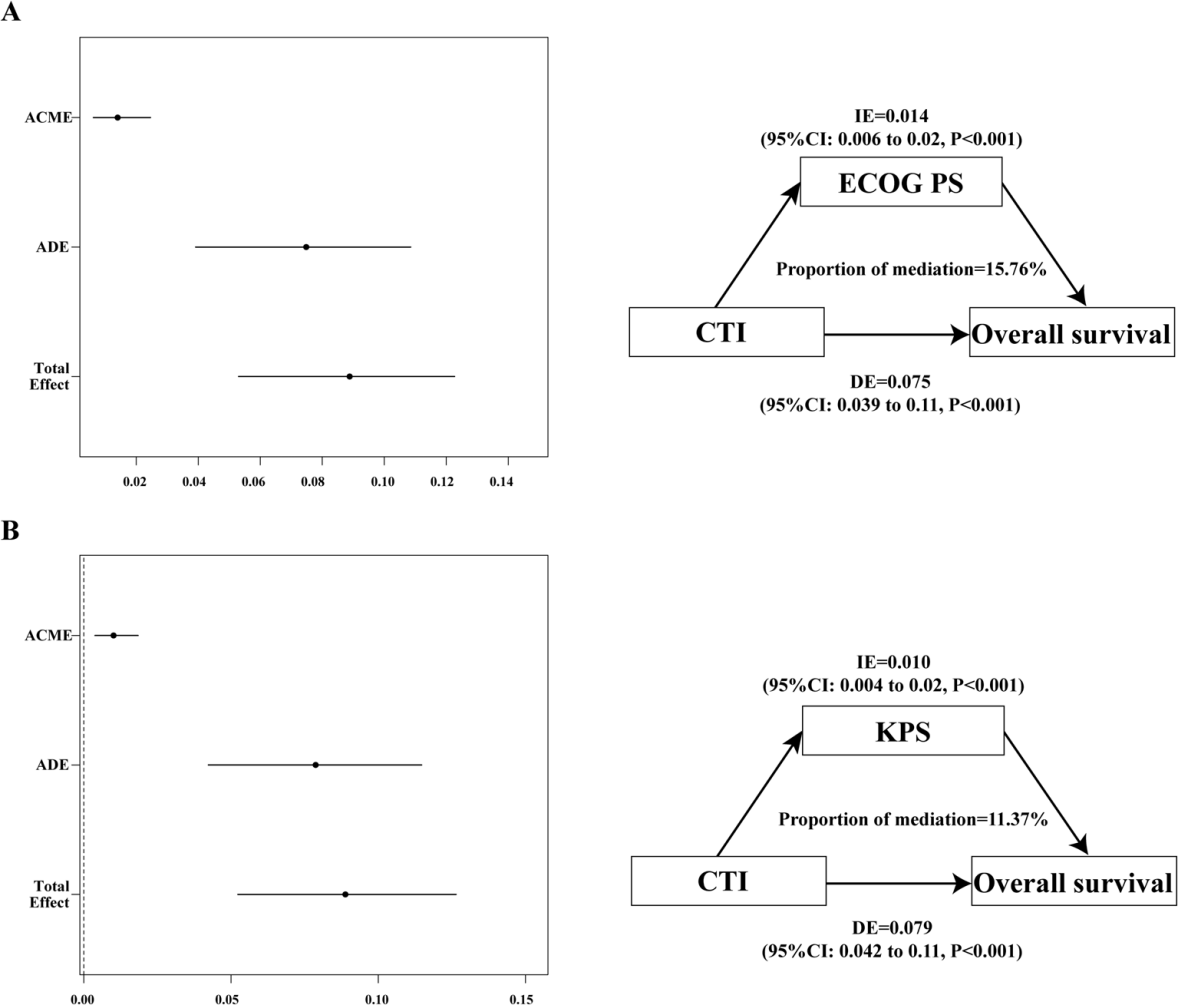
The mediation proportion of ECOG PS and KPS in CTI attributed to OS in patients with GI cancer. **(A)** Decompose the total association between CTI and OS into ECOG PS-mediated direct association, indirect association, and mediation proportion.; **(B)** Decompose the total association between CTI and OS into KPS-mediated direct association, indirect association, and mediation proportion. CTI, C-reactive protein-triglyceride glucose index; ECOG PS, eastern cooperative oncology group performance status; KPS, karnofsky performance status; OS, overall survival; ACME, average causal mediation effects (indirect effect); ADE, average direct effects; IE, indirect effect; DE, direct effect.

The physical activity of patients may have an impact on survival; therefore, we speculated that quality of life may mediate the poor prognosis based on the CTI. The mediating effect analysis showed that the mediating ratios of KPS and ECOG PS were 11.4% and 15.8%, respectively (**Figure 5**).

#### Association of the CTI with the KPS, ECOG PS, and 90-day and 180-day mortalities

We analyzed the correlation of the CTI with the KPS, ECOG PS, and 90-day and 180-day mortalities. There was a significant positive correlation of the CTI with the KPS (OR=2.54, 95% CI=1.40–4.69), ECOG PS (OR=1.94, 95% CI=1.21–3.13), and 90-day (OR=3.25, 95% CI=1.75–6.23), and 180-day mortalities (OR=2.66, 95% CI=1.72–4.15) (**Supplementary Table S3**).

#### Compare the prognostic abilities of CTI and common indicators in patients with gastrointestinal cancer

We compared the prognostic ability of CTI and other 7 common prognostic markers in patients with gastrointestinal cancer. The prognostic AUC results showed that CTI has advantages over other prognostic indicators (**Supplementary Figure S6**).

## Discussion

In the training, internal validation, and external validation cohorts, the CTI strongly predicted short- and long-term survival. The CTI can not only reflect SI and IR but can also predict survival outcomes in patients with GI cancer. Importantly, we also found that the CTI is positively correlated with 90-day and 180-day mortalities in patients with GI cancer. The CTI is also associated with recent survival outcomes in patients with GI cancer and may be associated with high inflammation and IR status. Some studies have found that an increase in hypersensitivity to CRP or IR is associated with increased mortality in patients (9). This can be explained by the composition of the index or by other factors. The role of inflammation in tumorigenesis has been widely accepted. Tumor cells can cause inflammation and increase serum CRP levels. Moreover, CRP is part of the host immune response to tumor cells, reflecting the inflammatory state of the body (35). Epidemiological studies indicated that CRP is correlated to increased risks of malignant tumors, anorexia-cachexia syndrome, and poor survival, including tumor recurrence, tumor size, lymph node metastasis, and distant metastasis (36, 37). Cytokines and chemokines released by tumor-infiltrating immune cells and tumor cells can activate inflammatory responses, create favorable conditions for tumor growth, induce DNA damage, promote angiogenesis, and promote tumor spread and metastasis (38). The TyG index reflects the role of cytotoxicity and glucotoxicity in IR. Cancer cells have more demand for glucose than normal cells, which can lead to hypoglycemia and stimulate the increase of glucagon. Hyperinsulinemia itself can induce an increase in inflammation, thus promoting IR (39, 40). Some studies have shown that IR activation can promote the MAP/ERK and PI3K/Akt/mTOR pathways, resulting in adverse cell progression (41).

We found that a higher CTI in older patients than in younger patients, and this phenomenon is consistent with the clinical phenomenon we have observed. With an increase in age, the level of inflammation increases; inflammatory aging is obvious, and an increase in inflammation may cause an increase in IR. CRP can be used as an *in vivo* marker of exposure to reflect the aging state of the body (42). Aging people are susceptible to disease, and the older they are, the weaker their body’s ability to resist disease, especially in cancer patients. Studies have shown that IR levels increase with age (43). Compared with young individuals, insulin-stimulated glucose uptake in isolated adipocytes of middle-aged adults is impaired (43). This may be correlated to abnormal glucose metabolism in the patient. We speculate that inflammation and IR increase with age in patients with GI cancer.

Considering the heterogeneity of upper and lower GI cancers, we performed the prognostic value of the CTI in patients with upper and lower digestive tract cancers, respectively, and the results were statistically different. However, we found that patients with lower digestive tract cancer with a high CTI had a higher mortality risk than those with upper GI cancers. We speculate that compared with patients with upper GI cancers, those with lower GI cancer are more likely to have intestinal obstruction, which can lead to a reduced diet, malnutrition, or cancer cachexia. The reduced diet caused by anorexia is largely caused by inflammation, and the state of cachexia is correlated to the SI and IR state of the patient; thus, it may lead to a high CTI and high mortality risk in patients with lower digestive tract cancer.

We found that patients with GI patients with a high CTI had poorer physical activity than those with a low CTI, and the intermediary analysis supported our results. Furthermore, we analyzed the relationship between the CTI and poor quality of life and found a significant positive correlation. Increased physical activity can improve glucose tolerance, reduce IR and SI, and reduce the risk of cancer (44, 45). The mediating effect mediated by quality of life may be related to the status of SI and IR in patients, which may bring unexpected results to patients by improving their physical activity.

Subgroup analysis revealed a significant interaction between high CTI, surgery, and chemotherapy. Some studies found that patients with EC have a low survival rate after surgical treatment, which may be related to core muscle atrophy (46). Core muscle atrophy is associated with tumor load, inflammation, and IR. Moreover, muscle atrophy is a cancer marker, which may indicate that patients are more likely to experience cachexia. In clinical practice, we have found that inflammatory levels and blood glucose are elevated in patients with cancer, which may be related to the poor survival of patients with a high CTI after surgery. When receiving chemotherapy, patients with cancer may experience vomiting and anorexia, which in turn induce weight loss and even malnutrition, both of which are associated with inflammation and IR; however, these symptoms may improve at the end of chemotherapy.

This study had some limitations. First, there was heterogeneity in the different types of GI cancer, and our findings need to be analyzed and verified in other cohorts. Second, the CTI cutoff value we selected for GI cancer may be different from other cutoff points, which is related to the nature of the patients with GI cancer themselves. Our cutoff value was based on patients from multiple medical centers in China, and the cutoff value still needs to be further verified. Finally, this was a cross-sectional study, so we need to consider a longitudinal analysis in a future collection of data, such as changes in patients receiving treatment including surgery, radiotherapy, and chemotherapy.

## Conclusions

We demonstrated that the CTI is related to SI and IR and that it is an effective indicator for predicting short- and long-term survival outcomes in patients with GI cancer. A high CTI is associated with poor survival outcomes in patients with GI cancer. Additionally, the CTI was associated with the age of patients with GI cancer and 90-day and 180-day mortalities. The CTI is expected to be a practical tool for short- and long-term prognostic assessment in patients with GI cancer.

## Data Availability

The raw data supporting the conclusions of this article will be made available by the authors, without undue reservation.

## Supplementary material

Supplementary Figure 1 Flowchart of patient selection for this study. Notes: GI, gastrointestinal; EC, esophagus cancer; GC, gastric cancer; CRC, colorectal cancer.

Supplementary Figure 2 Correlation between CTI and components (CRP and TyG). (A) Different age groups; (B) Different sex groups. Notes: CTI, C-reactive protein-triglyceride glucose index; CRP: C-reactive protein; TyG: triglyceride-glucose index.

Supplementary Figure 3 The optimal cut-off values of CTI in patients with GI cancer. Notes: CTI, C-reactive protein-triglyceride glucose index.

Supplementary Figure 4 The calibration curves of CTI in the different cohorts of patients with GI cancer. (A-C) 1-, 3-, and 5-year calibration curves of CTI in the training cohort; (D-F) 1-, 3-, and 5-year calibration curves of CTI in the internal validation cohort; (G-I) 1-, 3-, and 5-year calibration curves of CTI in the external validation cohort. Notes: GI, gastrointestinal; CTI, C-reactive protein-triglyceride glucose index; OS, overall survival.

Supplementary Figure 5 The restricted spline curves of CTI in the different cohorts of patients with GI cancer. (A-C) The restricted spline curve of CTI in the training cohort; (D-F) The restricted spline curve of CTI in the internal validation cohort; (G-I) The restricted spline curve of CTI in the external validation cohort. Notes: GI, gastrointestinal; CTI, C-reactive protein-triglyceride glucose index; OS, overall survival.

Supplementary Figure 6 The prognostic curve of CTI in the training cohort of patients with GI cancer. Notes: GI, gastrointestinal; CTI, C-reactive protein-triglyceride glucose index; OS, overall survival.
