# Supplementary figures and images for: Prognostic importance of an indicator related to systemic inflammation and insulin resistance in patients with gastrointestinal cancer: a prospective study
Source: Front Oncol. 2024 Dec 2;14:1394892. doi: 10.3389/fonc.2024.1394892 (PMC11646804; doi:10.3389/fonc.2024.1394892)

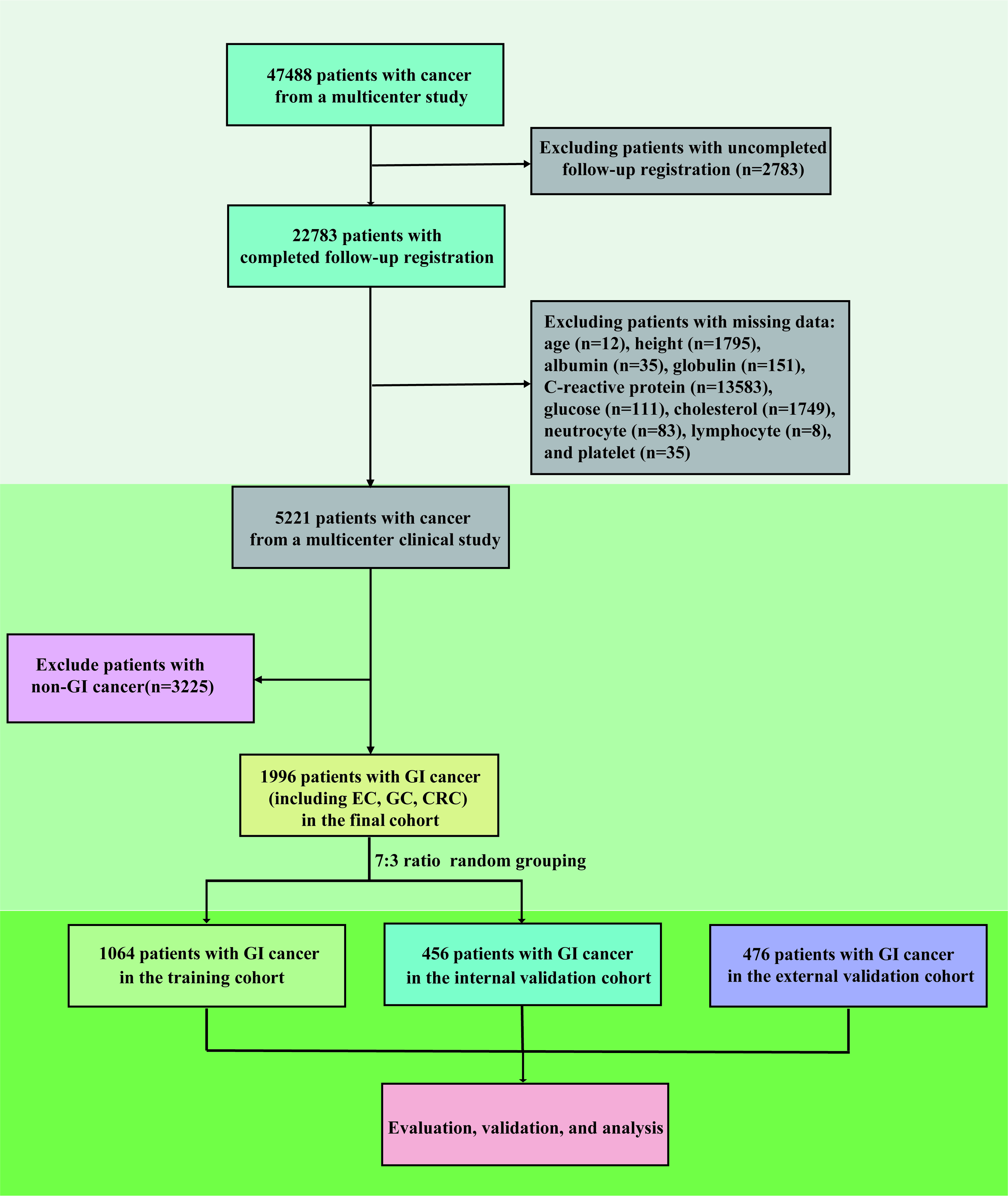

Supplement: Supplementary Figure 1 — Flowchart of patient selection for this study. Notes: GI, gastrointestinal; EC, esophagus cancer; GC, gastric cancer; CRC, colorectal cancer. [file Image1.tif]

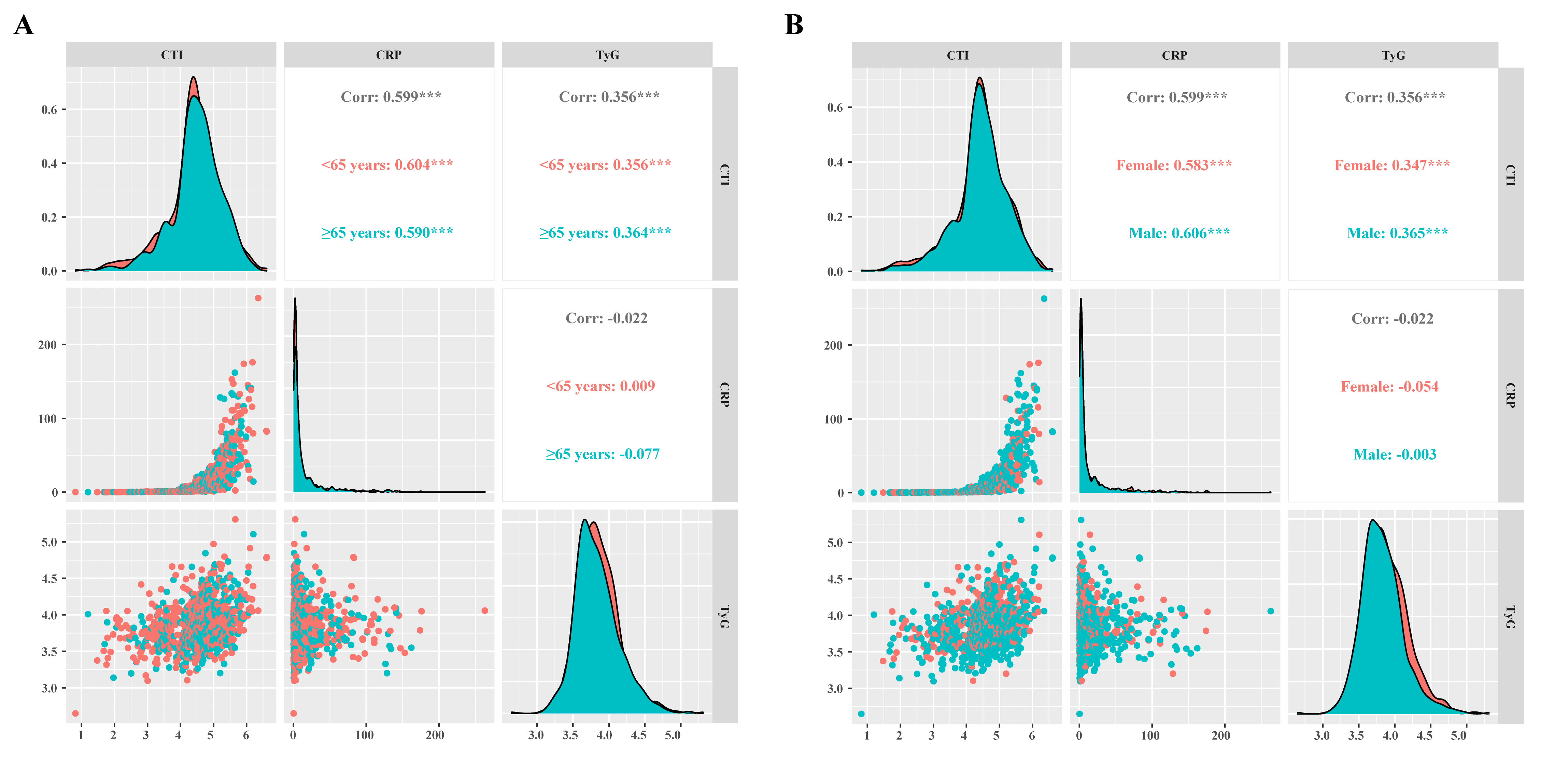

Supplement: Supplementary Figure 2 — Correlation between CTI and components (CRP and TyG). (A) Different age groups; (B) Different sex groups. Notes: CTI, C-reactive protein-triglyceride glucose index; CRP: C-reactive protein; TyG: triglyceride-glucose index. [file Image2.tif]

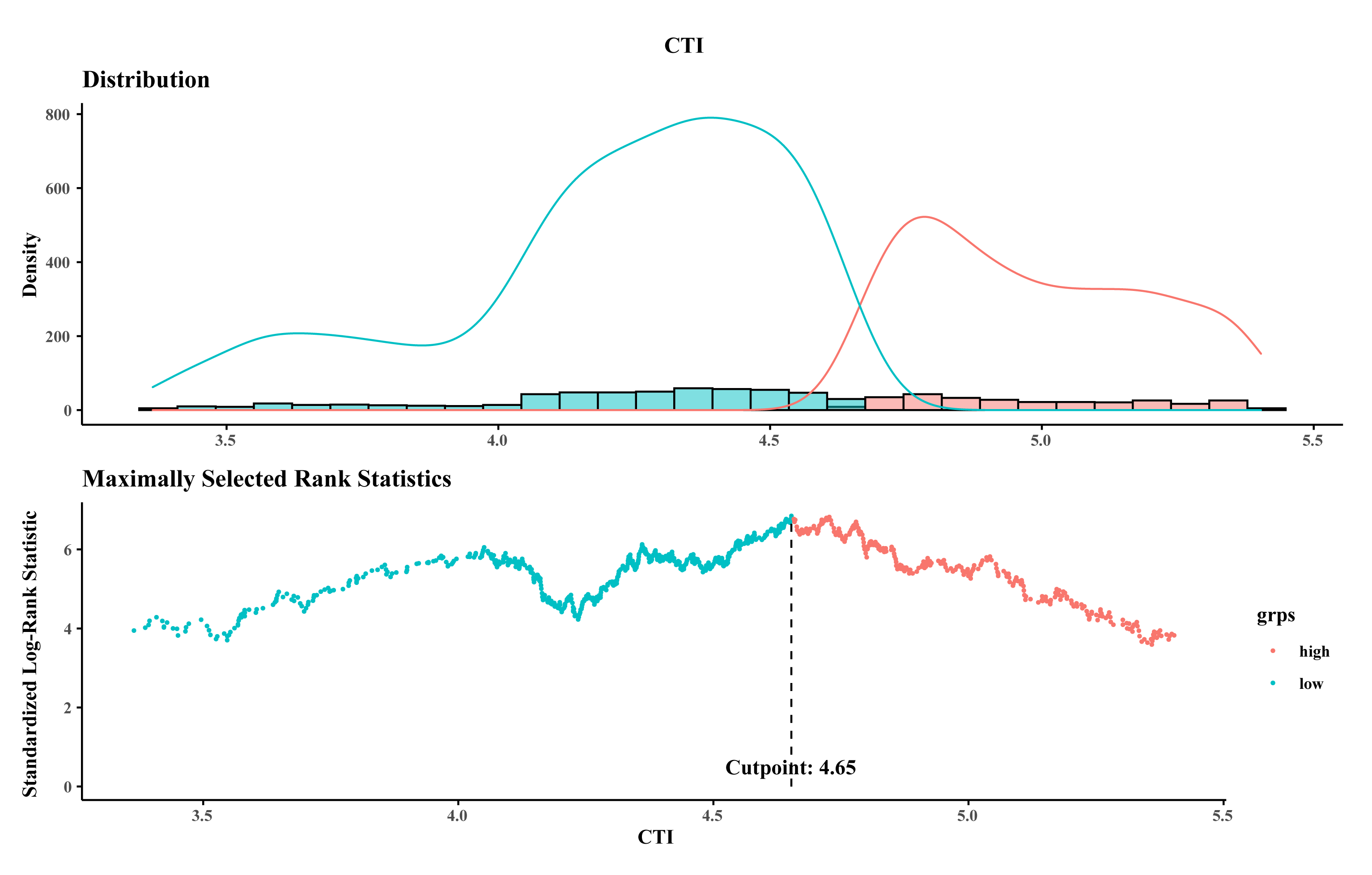

Supplement: Supplementary Figure 3 — The optimal cut-off values of CTI in patients with GI cancer. Notes: CTI, C-reactive protein-triglyceride glucose index. [file Image3.tif]

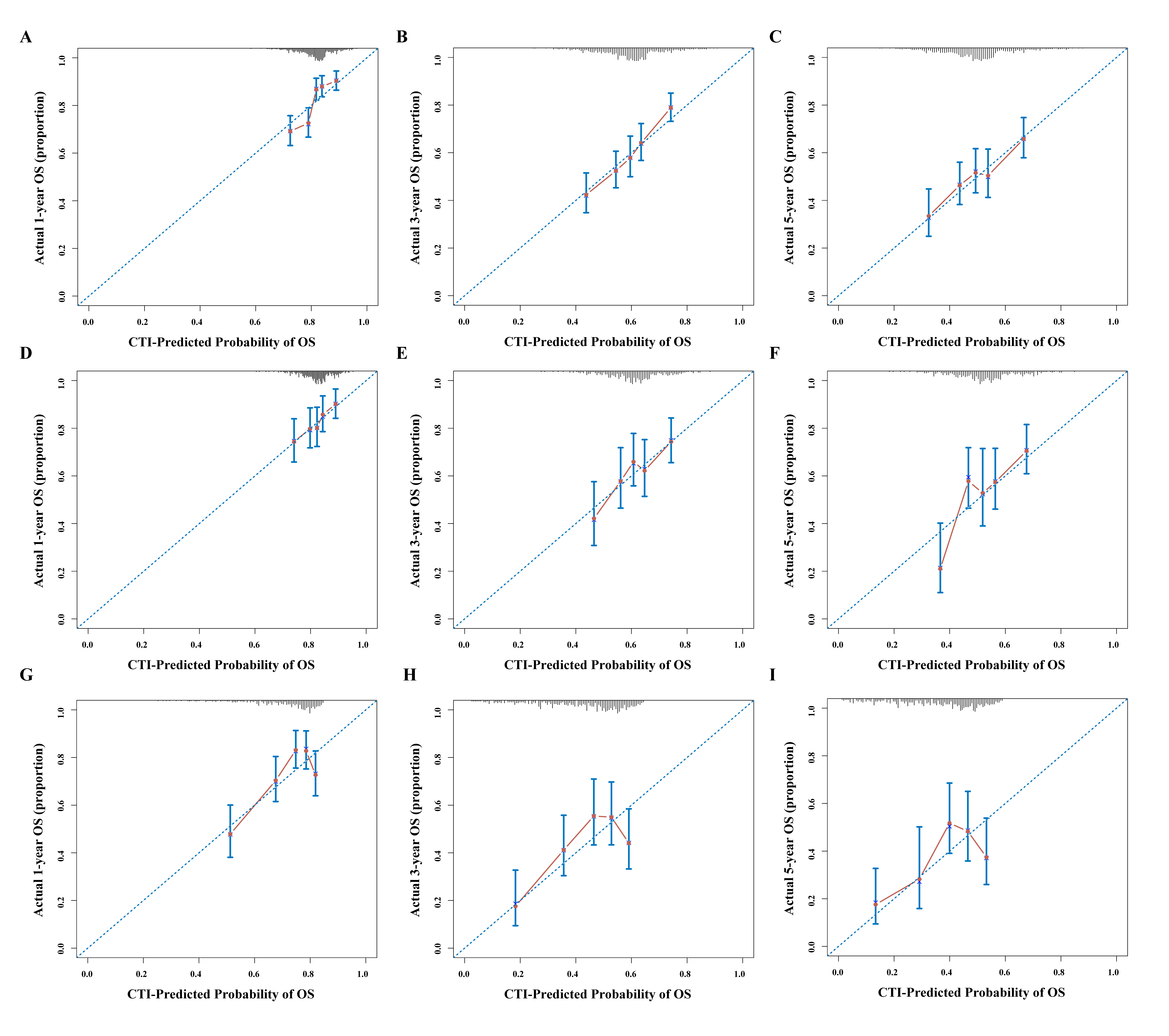

Supplement: Supplementary Figure 4 — The calibration curves of CTI in the different cohorts of patients with GI cancer. (A-C) 1-, 3-, and 5-year calibration curves of CTI in the training cohort; (D-F) 1-, 3-, and 5-year calibration curves of CTI in the internal validation cohort; (G-I) 1-, 3-, and 5-year calibration curves of CTI in the external validation cohort. Notes: GI, gastrointestinal; CTI, C-reactive protein-triglyceride glucose index; OS, overall survival. [file Image4.tif]

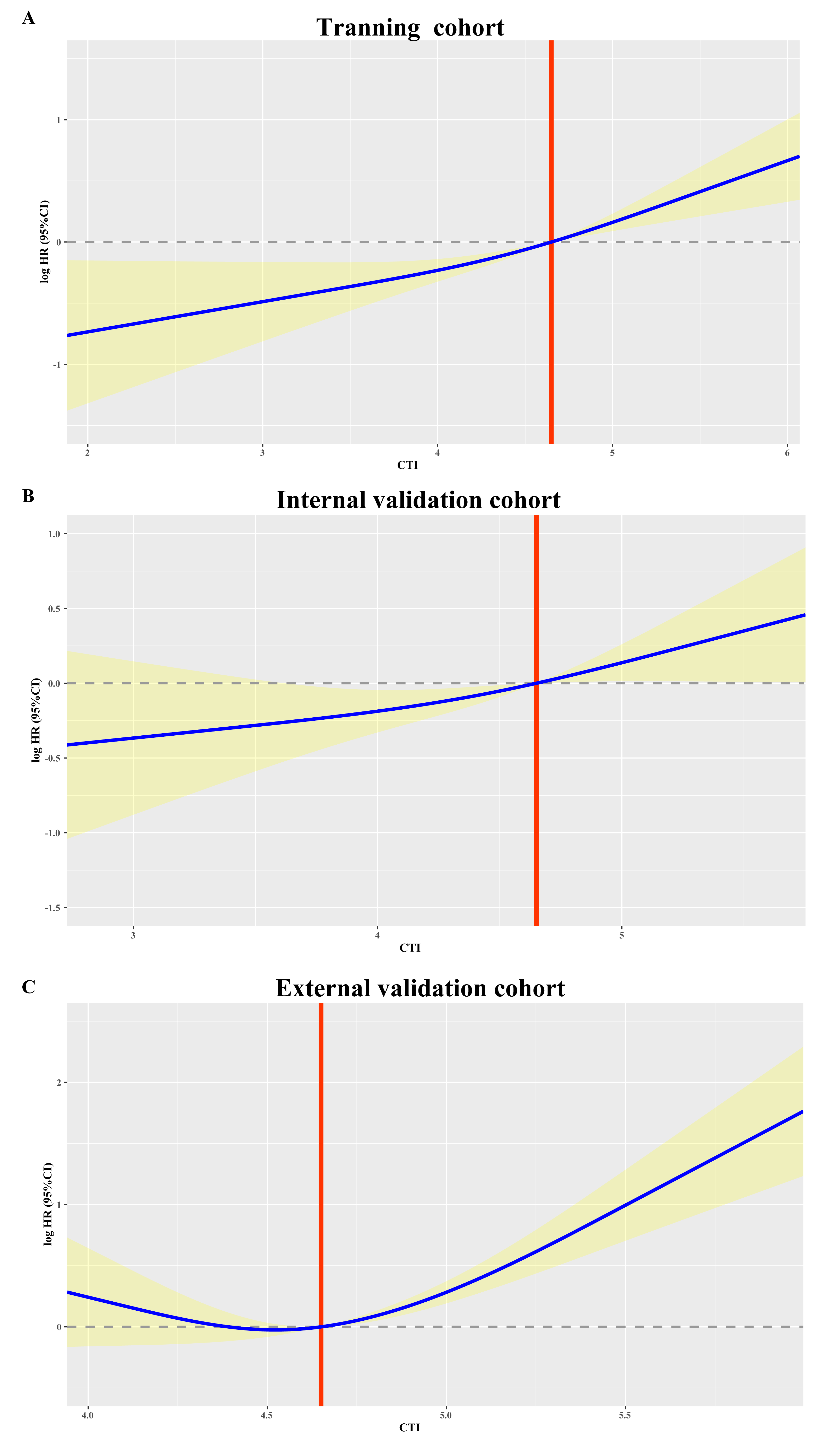

Supplement: Supplementary Figure 5 — The restricted spline curves of CTI in the different cohorts of patients with GI cancer. (A-C) The restricted spline curve of CTI in the training cohort; (D-F) The restricted spline curve of CTI in the internal validation cohort; (G-I) The restricted spline curve of CTI in the external validation cohort. Notes: GI, gastrointestinal; CTI, C-reactive protein-triglyceride glucose index; OS, overall survival. [file Image5.tif]

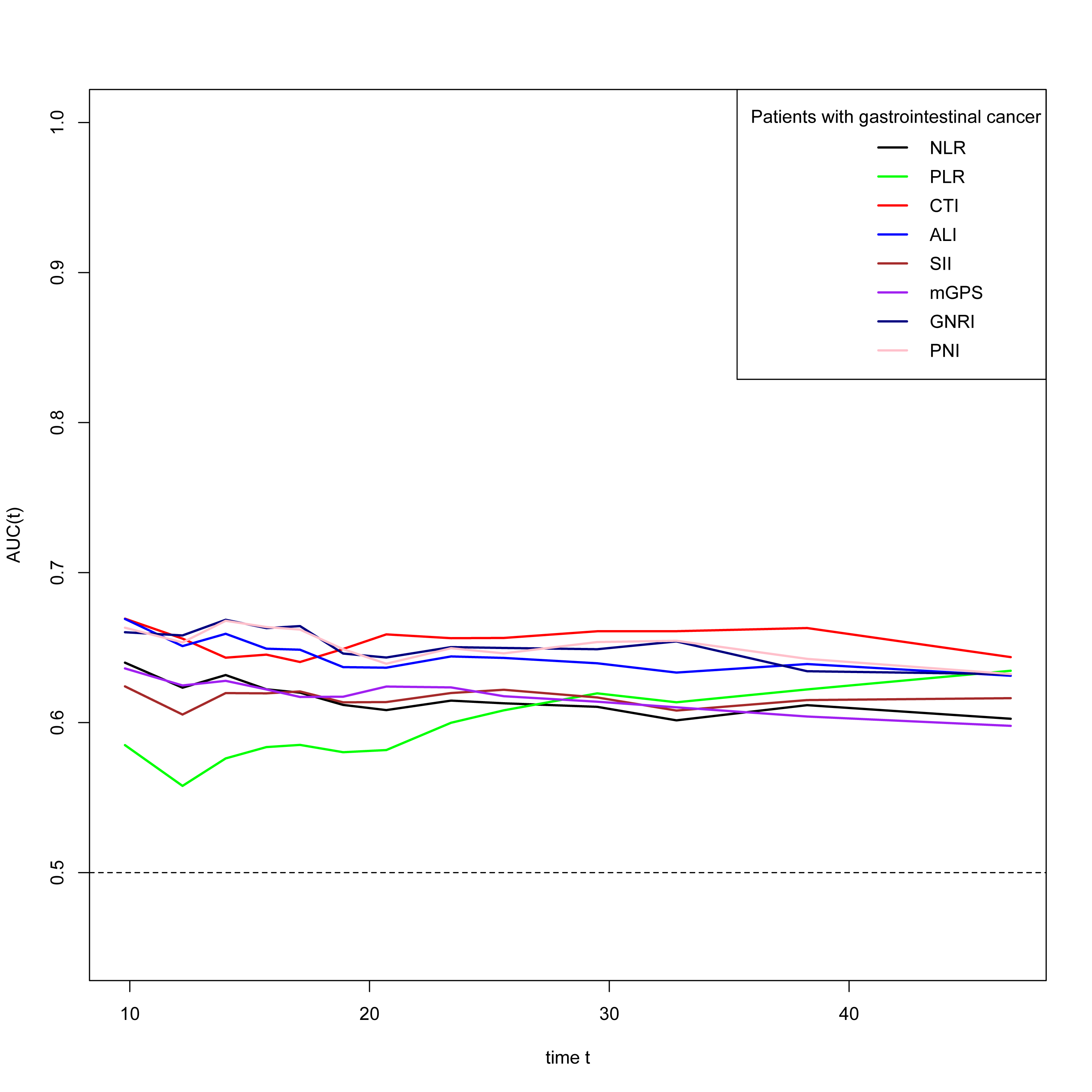

Supplement: Supplementary Figure 6 — The prognostic curve of CTI in the training cohort of patients with GI cancer. Notes: GI, gastrointestinal; CTI, C-reactive protein-triglyceride glucose index; OS, overall survival. [file Image6.tif]
