# Supplementary material for: Prognostic importance of an indicator related to systemic inflammation and insulin resistance in patients with gastrointestinal cancer: a prospective study
Source: Front Oncol. 2024 Dec 2;14:1394892. doi: 10.3389/fonc.2024.1394892 (PMC11646804; doi:10.3389/fonc.2024.1394892)
Supplement: Supplementary file 7 [file Table1.docx]

**Table S1 Baseline characteristics stratified by CTI in the training cohort.**

| Variables | CTI<4.65  (n=663) | CTI≥4.65  (n=401) | P value |
| --- | --- | --- | --- |
| Sex (%) |  |  |  |
| male | 448 (67.6) | 276 (68.8) | 0.720 |
| female | 215 (32.4) | 125 (31.2) |  |
| Age, years (mean (SD)) | 59.52 (11.41) | 62.43 (10.94) | <0.001 |
| BMI, kg/m^2 (mean (SD)) | 21.64 (3.39) | 22.20 (3.71) | 0.012 |
| BMI, kg/m^2 (%) |  |  |  |
| <24 | 499 (75.3) | 275 (68.6) | 0.021 |
| ≥24 | 164 (24.7) | 126 (31.4) |  |
| Smoking，yes (%) | 283 (42.7) | 197 (49.1) | 0.047 |
| Drinking, yes (%) | 176 (26.5) | 114 (28.4) | 0.55 |
| Diabetes, yes (%) | 42 (6.3) | 63 (15.7) | <0.001 |
| Hypertension, yes (%) | 109 (16.4) | 109 (27.2) | <0.001 |
| CHD, yes (%) | 27 (4.1) | 17 (4.2) | 1.000 |
| Tumor stage (%) |  |  |  |
| Ⅰ-Ⅱ | 199 (30.0) | 87 (21.7) | 0.004 |
| Ⅲ-Ⅳ | 464 (70.0) | 314 (78.3) |  |
| Surgery, yes (%) | 447 (67.4) | 229 (57.1) | 0.001 |
| Radiotherapy, yes (%) | 47 (7.1) | 39 (9.7) | 0.158 |
| Chemotherapy, yes (%) | 426 (64.3) | 253 (63.1) | 0.752 |
| Tumor type (%) |  |  | 0.316 |
| Upper digestive tract tumors | | | |
| EC (%) | 262 (39.5) | 140 (34.9) |  |
| GC (%) | 109 (16.4) | 73 (18.2) |  |
| Lower digestive tract tumors | | | |
| CRC (%) | 292 (44.0) | 188 (46.9) |  |
| Glucose, mmol/L (mean (SD)) | 5.43 (1.18) | 6.32 (2.25) | <0.001 |
| TG, mmol/L (mean (SD)) | 1.23 (0.58) | 1.72 (1.48) | <0.001 |
| CRP, mg/L (median (IQR)) | 2.60 (2.37) | 20.00 (334.00) | <0.001 |
| TyG (mean (SD)) | 3.78 (0.25) | 3.97 (0.32) | <0.001 |
| CTI (mean (SD)) | 3.98 (0.65) | 5.17 (0.40) | <0.001 |
| ECOG (%) |  |  |  |
| <2 | 615 (92.8) | 324 (80.8) | <0.001 |
| ≥2 | 48 (7.2) | 77 (19.2) |  |
| KPS (mean (SD)) | 87.00 (11.06) | 80.52 (16.58) | <0.001 |
| PGSGA (mean (SD)) | 6.65 (4.42) | 8.97 (5.83) | <0.001 |
| Nutrition intervention (%) | 186 (28.1) | 170 (42.4) | <0.001 |
| TSF, cm (mean (SD)) | 14.56 (9.23) | 15.12 (9.85) | 0.350 |
| LOS, days (mean (SD)) | 11.70 (11.56) | 12.58 (9.51) | 0.198 |
| Hospital costs, yuan (mean (SD)) | 31385.75 (59127.06) | 33961.24 (66506.91) | 0.512 |

Notes: BMI: body mass index; CHD, coronary heart disease; EC, esophagus cancer; GC, gastric cancer; CRC, colorectal cancer; TG, triglyceride; CTI, CRP-TyG index; CRP, C-reactive protein; TyG: triglyceride-glucose index; KPS, karnofsky performance status; ECOG PS: eastern cooperative oncology group performance status; PGSGA, Patient Generated Subjective Global Assessment; TSF, triceps skinfold thickness; LOS, length of stay.
