# Supplementary material for: Prognostic importance of an indicator related to systemic inflammation and insulin resistance in patients with gastrointestinal cancer: a prospective study
Source: Front Oncol. 2024 Dec 2;14:1394892. doi: 10.3389/fonc.2024.1394892 (PMC11646804; doi:10.3389/fonc.2024.1394892)
Supplement: Supplementary file 8 [file Table2.docx]

**Table S2 Sensitivity analysis***

| Variables | OS (model 1) | | OS (model 4) | |
| --- | --- | --- | --- | --- |
|  | Crude HR(95%CI) | Crude P | Adjusted HR(95%CI) | Adjusted P |
| as continues (per SD) | 1.29 (1.14-1.45) | <0.001 | 1.18 (1.04-1.34) | 0.013 |
| By cut-off |  |  |  |  |
| CTI<4.65 | ref. |  | ref. |  |
| CTI≥4.65 | 1.7 (1.34-2.15) | <0.001 | 1.43 (1.1-1.85) | 0.007 |
| By Interquartile |  |  |  |  |
| Q1(<4.11) | ref. |  | ref. |  |
| Q2(4.11-4.46) | 1.68 (1.19-2.37) | 0.003 | 1.41 (1.00-2.00) | 0.051 |
| Q3(4.46-4.91) | 1.69 (1.20-2.40) | 0.003 | 1.58 (1.10-2.27) | 0.013 |
| Q4(>4.91) | 2.17 (1.54-3.06) | <0.001 | 1.65 (1.14,2.39) | 0.008 |

Notes: * The sensitivity analysis was to exclude patients who died within 6 months. OS, overall survival; HR, hazards ratio; CI, confidence interval; CTI, CRP-TyG index; CRP, C-reactive protein; TyG: triglyceride-glucose index; BMI: body mass index; KPS, karnofsky performance status; EORTC QLQ-C30, The European Organization for Research and Treatment of Cancer (EORTC), Quality of Life Questionnaire-Core 30 (QLQ-C30); ECOG PS: eastern cooperative oncology group performance status; PGSGA, Patient Generated Subjective Global Assessment; TSF, triceps skinfold thickness.

Model 1: Unadjusted.

Model 4: Adjusted for age, sex, BMI, tumor stage, tumor types, surgery, chemotherapy, radiotherapy, smoking status, alcohol consumption, KPS, ECOG PS, PGSGA, nutrition intervention, diabetes, hypertension, coronary heart disease, and TSF.
