# Supplementary material for: Prognostic importance of an indicator related to systemic inflammation and insulin resistance in patients with gastrointestinal cancer: a prospective study
Source: Front Oncol. 2024 Dec 2;14:1394892. doi: 10.3389/fonc.2024.1394892 (PMC11646804; doi:10.3389/fonc.2024.1394892)
Supplement: Supplementary file 9 [file Table3.docx]

**Table S3 Logistic regression analysis.**

| Variables | Crude OR(95%CI)* | Crude P | Adjusted OR(95%CI)# | Adjusted P |
| --- | --- | --- | --- | --- |
| **90-day mortality** | | | | |
| CTI<4.65 | ref. |  | ref. |  |
| CTI≥4.65 | 4.21(2.39-7.72) | <0.001 | 3.25(1.75-6.23) | <0.001 |
| **180-day mortality** | | | | |
| CTI<4.65 | ref. |  | ref. |  |
| CTI≥4.65 | 3.24(2.17-4.88) | <0.001 | 2.66(1.72-4.15) | <0.001 |
| **KPS (≤60)** | | | | |
| CTI<4.65 | ref. |  | ref. |  |
| CTI≥4.65 | 4.23(2.64-6.93) | <0.001 | 2.54(1.40-4.69) | 0.002 |
| **ECOG PS (≥2)** | | | | |
| CTI<4.65 | ref. |  | ref. |  |
| CTI≥4.65 | 3.04(2.08-4.50) | <0.001 | 1.94(1.21-3.13) | 0.006 |

Notes: OR, odd ratio; CI, confidence interval; CTI, CRP-TyG index; CRP, C-reactive protein; TyG: triglyceride-glucose index; BMI: body mass index; KPS, karnofsky performance status; ECOG PS: eastern cooperative oncology group performance status; PGSGA, Patient Generated Subjective Global Assessment; TSF, triceps skinfold thickness.

*Model 1: Unadjusted.

#Model 4: Adjusted for age, sex, BMI, tumor stage, tumor types, surgery, chemotherapy, radiotherapy, smoking status, alcohol consumption, KPS, ECOG PS, PGSGA, nutrition intervention, diabetes, hypertension, coronary heart disease, and TSF.
